# Visceral fat and cardiorespiratory fitness with prevalence of pre-diabetes/diabetes mellitus among middle-aged and elderly Japanese people: WASEDA’S Health Study

**DOI:** 10.1371/journal.pone.0241018

**Published:** 2020-10-20

**Authors:** Chiyoko Usui, Ryoko Kawakami, Kumpei Tanisawa, Tomoko Ito, Hiroki Tabata, Satoshi Iizuka, Takuji Kawamura, Taishi Midorikawa, Susumu S. Sawada, Suguru Torii, Shizuo Sakamoto, Katsuhiko Suzuki, Kaori Ishii, Koichiro Oka, Isao Muraoka, Mitsuru Higuchi

**Affiliations:** 1 Faculty of Sport Sciences, Waseda University, Saitama, Japan; 2 Waseda Institute for Sport Sciences, Waseda University, Saitama, Japan; 3 Sportology Center, Juntendo University Graduate School of Medicine, Tokyo, Japan; 4 Department of Sport Sciences, Japan Institute of Sports Sciences, Tokyo, Japan; 5 College of Health and Welfare, J. F. Oberlin University, Tokyo, Japan; Ritsumeikan University, JAPAN

## Abstract

The relationships between cardiorespiratory fitness (CRF) measurements not confounded by adiposity and the prevalence of pre-diabetes mellitus (pre-DM) and diabetes mellitus (DM) are not well known. Thus, we aimed to investigate the associations of visceral fat (VF) and CRF with the prevalence of pre-DM/DM among Japanese adults. The study included 970 individuals (327 women and 643 men) who were 40–87 years old and had complete health examinations, abdominal fat area, and fitness data from WASEDA’S Health Study during 2015–2018. The VF area was measured using magnetic resonance imaging. CRF was measured using a cycle ergometer and was defined as VO_2_peak divided by fat free mass. The pre-DM/DM was identified based on the questionnaire and fasting blood tests. The odds ratios (ORs) and 95% confidence intervals (CIs) for prevalence of pre-DM/DM were calculated. Seventy-three participants had pre-DM and 48 participants had DM. Compared to the low VF group, the high VF group had a higher prevalence of pre-DM/DM (OR: 1.87, 95% CI: 1.18–2.96), although no significant relationship was observed between CRF and pre-DM/DM prevalence (*P* for trend = 0.239). The sub-group analyses also revealed no significant relationship between CRF and pre-DM/DM prevalence in the low VF group (*P* for trend = 0.979), although CRF values were inversely related to the prevalence of pre-DM/DM in the high VF group (*P* for trend = 0.024). Although CRF was not independently related to the prevalence of pre-DM/DM after adjusting for adiposity, higher VF values were related to a higher prevalence of pre-DM/DM. In addition, CRF levels were inversely associated with the prevalence of pre-DM/DM only among high VF individuals.

## Introduction

The increasing prevalence of diabetes mellitus (DM) is becoming a global public health issue. The International Diabetes Federation estimated that 1 in 11 adults who were 20–79 years old during 2019 had DM (463 million adults) and predicted that this number will reach 700 million people by 2045 [1]. During recent years, DM has rapidly become widespread in Asia [1, 2], which is related to obesity, a sedentary lifestyle, physical inactivity, energy-dense diets, and population aging in Asia [2, 3]. In particular, it has been reported that the increasing prevalence of obesity parallels the increasing prevalence of DM [2, 4, 5]. Furthermore, Asian individuals generally have poorer β-cell function compared to Western individuals and they develop DM at a younger age and lower body mass index (BMI) [2, 6–8]. High levels of abdominal (or central) adiposity, which can increase the risk of DM, are extremely common among Asian individuals [8], who generally have higher visceral adiposity than BMI-matched individuals of European descent [7, 9, 10].

Abdominal adiposity can be evaluated using waist circumference, waist-to-hip ratio, subcutaneous fat (SF) area, and visceral fat (VF) area, with VF being an independent predictor of the development of DM and insulin resistance [11, 12]. Cardiorespiratory fitness (CRF) is an objective index of habitual physical activity that is assessed using either maximum oxygen uptake (VO_2_max) or peak oxygen uptake (VO_2_peak), and low CRF is also a risk factor for the development of DM and insulin resistance [13–16].

Previous research has indicated that the risks of DM and insulin resistance can be influenced by a combination of increased abdominal adiposity and decreased CRF [12, 17, 18]. We have reported that abdominal adiposity (VF and SF), but not CRF, contributes to insulin resistance among non-diabetic individuals [12]. Furthermore, Racette et al. [17] highlighted the importance of abdominal adiposity, which they evaluated using waist circumference, as a risk factor for insulin resistance among older people, as waist circumference exhibited the strongest relationship with insulin resistance. However, they also reported that CRF was a significant predictor of insulin resistance among older people, albeit a less robust predictor than abdominal adiposity. Thus, there have been conflicting findings regarding the relationships between CRF and the development of DM and insulin resistance. Nevertheless, previous studies may have been confounded by adiposity when evaluating CRF, as the analyses were based on VO_2_peak (or VO_2_max) per body weight, which underestimates the CRF of obese individuals [19, 20]. This is because the fat mass has little effect on VO_2_peak values and is already included in body weight. Therefore, evaluating CRF based on VO_2_peak/body weight may provide inaccurate results regarding the relationships between CRF and the development of DM and insulin resistance. Dividing VO_2_peak by fat free mass (FFM), which includes the internal organs (e.g., heart and liver) and skeletal muscle, leads to a qualitative assessment of aerobic capacity of the metabolically active tissue [21, 22]. In addition, previous reports have highlighted the physiological superiority of VO2peak per FFM as a measure of CRF compared to VO2peak/body weight in the prediction of clinical outcomes such as chronic heart failure and mortality risk [23, 24]. Imboden et al. [24] suggested that VO2peak per FFM is not only the most accurate measure of CRF as it relates to exercise performance but also the best way to present CRF as it pertains to health.

A previous study used VO_2_max divided by FFM and revealed that insulin sensitivity in middle-aged men without DM was inversely correlated to waist circumference (r = –0.71) and positively correlated to VO_2_max/FFM (r = 0.78) [25]. Moreover, a multiple linear regression analysis performed by Jukarainen et al. [26] found weak associations between the VO_2_max/FFM of healthy adult volunteers and insulin sensitivity (β = 0.10) or insulin resistance (β = –0.15). Therefore, the reported relationships between CRF measures not confounded by obesity and DM are inconsistent, although no studies have comprehensively evaluated this relationship after adjusting for VF. The present study aimed to evaluate whether VF or CRF were independently related to the prevalence of pre-DM/DM, after adjusting for adiposity, among Japanese individuals. This information may clarify whether the relationship between CRF and pre-DM/DM is related to differences in VF values, which is thought to be an important factor that influences the increased prevalence of pre-DM/DM.

## Methods

### Participants

This cross-sectional study evaluated baseline survey data from the Waseda Alumni's Sports, Exercise, Daily Activity, Sedentariness and Health Study (WASEDA'S Health Study). The WASEDA’S Health Study is a cohort study that surveys the relationship between sports/exercise/physical activity/sedentary behavior and health outcomes among alumni of Waseda University and their spouses who are ≥40 years old. The study included four cohorts (cohorts A–D). The present study evaluated participants in the cohort D of WASEDA’S Health Study, who underwent detailed physical fitness and health examinations on campus.

Between March 2015 and November 2018, a total of 1,168 individuals participated in the cohort D of WASEDA’S Health Study. The exclusion criteria for the present study were a history of heart disease or cancer (n = 104), participants who did not undergo magnetic resonance imaging or with unavailable data (n = 33), participants who did not undergo dual-energy X-ray absorptiometry (DXA, e.g., because of internal or external metal objects) (n = 27), participants who ate breakfast on the measurement day (n = 9), and participants who were excluded from the energy intake calculations using a brief-type self-administered diet history questionnaire (BDHQ; n = 8) [27]. In addition, the present study excluded individuals with missing responses on the self-administered questionnaire (n = 1), individuals who did not complete the submaximal exercise test (n = 5), individuals with missing blood test data (n = 6), Japanese individuals living abroad and non-Japanese individuals (n = 4), and individuals who were associated with this study (n = 1). The final study sample included 970 eligible participants (327 women and 643 men).

Before the baseline survey, all participants were informed regarding the details of the study and provided their written informed consent to participate. The study was approved by the Research Ethics Committee of Waseda University (2014-G002) and conducted in accordance with the Declaration of Helsinki. The report was prepared in accordance with the Strengthening the Reporting of Observational Studies in Epidemiology (STROBE) reporting guidelines for cohort studies.

### General health examination

Self-administered questionnaires were used to collect information regarding sex, age, history of DM, hypertension and dyslipidemia (yes or no), smoking habit (current smoker, former smoker, non-smoker), and menstrual status (yes or no). The BDHQ was used to collect information regarding energy intake and alcohol intake.

Height and body weight were measured using a stadiometer (YHS-200D; YAGAMI Inc., Nagoya, Japan) and a multifrequency bioelectrical impedance analysis analyzer (MC-980A; Tanita Corp., Tokyo, Japan) while the participants wore lightweight clothing and were barefooted. The BMI values were calculated as weight divided by height squared (kg/m^2^). All participants were instructed to fast overnight (over 12 hours) before the blood sample collection (venous blood samples), which was performed the next morning after confirming that the participants had not eaten. The blood biochemistry parameters were related to lipid metabolism (triglycerides, total cholesterol, high-density lipoprotein cholesterol, and low-density lipoprotein cholesterol) and glycometabolism (fasting blood glucose, glycated hemoglobin [HbA_1c_], and insulin). Resting systolic and diastolic blood pressure were measured at least twice using an automatic sphygmomanometer (HEM-7250-IT; Omron Healthcare Co., Ltd., Kyoto, Japan) with the participants in a seated position, and the mean value was used as the blood pressure value. The percentage of body fat was measured using DXA (Delphi A until December 2016 or Horizon A after January 2017; Hologic Inc., Marlborough, MA, USA), and FFM values were calculated using the body weight and percentage body fat values.

### Identifying pre-diabetes mellitus, diabetes mellitus, hypertension, and dyslipidemia

The presence of pre-DM, DM, hypertension, and/or dyslipidemia was identified based on the self-administered questionnaire results and biochemical examination results of blood. These were further verified by identifying the disease status based on the physicians’ diagnoses of DM, hypertension, and dyslipidemia regardless of whether or not the patients were taking medications. DM was considered present based on fasting blood glucose of ≥126 mg/dL (≥7.0 mmol/L) and/or HbA_1c_ of ≥6.5% (48 mmol/mol). However, given that only a small proportion of the participants had DM, we also considered pre-DM as an outcome. Pre-DM was considered present based on fasting blood glucose of ≥110 mg/dL and/or HbA_1c_ of ≥6.0%. The blood glucose and HbA_1c_ criteria for identifying pre-DM and DM were selected based on the Japan Diabetes Society guidelines [28]. Hypertension was identified based on systolic blood pressure of ≥140 mmHg and/or diastolic blood pressure of ≥90 mmHg. Dyslipidemia was identified based on triglycerides of ≥150 mg/dL, low-density lipoprotein cholesterol of ≥140 mg/dL, and/or high-density lipoprotein cholesterol of <40 mg/dL.

### Measuring abdominal adiposity

The VF and SF areas were measured using magnetic resonance imaging (Signa 1.5T; General Electric Co., Milwaukee, Wisconsin, USA) as described previously [29]. The imaging conditions included a T-1 weighted spin-echo and axial-plane sequence with a slice thickness of 10 mm, a repetition time of 480 ms, and an echo time of 8.8 ms. Cross-sectional images were obtained at the umbilical region and participants were asked to exhale then hold their breath for approximately 20 s during the scan to reduce respiratory motion artifacts. The magnetic resonance imaging data were transferred to a personal computer in the Digital Imaging and Communications in Medicine (DICOM) file format, and cross-sectional VF and SF areas at the umbilical region were determined using image-analysis software (Slice-o-matic 4.3 and 5.0 for Windows; Tomovision, Montreal, Canada). The magnetic resonance imaging scans and analyses of VF and SF areas at the umbilical region were performed by several investigators. To minimize interobserver variation, two investigators double-checked all data which revealed intra-observer variation coefficients of 0.34±2.47% and 1.52±6.31%.

### Measuring cardiorespiratory fitness

The VO_2_peak values were measured by the submaximal exercise test using a cycle ergometer (828E; Monark Exercise AB, Kingdom of Sweden). During a 3-min rest period, electrocardiography measurements were performed (ML-9000 and MLX-1000; Fukuda Denshi Co., Ltd., Tokyo, Japan). The male participants were then started at 30 W and the female participants were started at 15 W, with increases of 15 W per minute until the participant reached exhaustion. Expired gases, pulmonary VO_2_ and VCO_2_, were analyzed by a breath-by-breath method using an automated gas analysis system (AE310S and AE100i; Minato Medical Science Co., Ltd., Osaka, Japan). A blood pressure cuff was also attached to the left upper arms to evaluate blood pressure using an automatic blood pressure monitor for submaximal exercise test (Tango M2; Sun Tech Medical Inc., Morrisville, North Carolina, USA). Safety evaluations were based on heart rate and blood pressure values from before and during the exercise.

The endpoints for the submaximal exercise test were the number of heart beats that reached approximately 90% of the age-predicted maximal heart rate (equal to 220—age), achieving a respiratory exchange ratio (RER) > 1.1, and rating of perceived exertion (RPE) of ≥18 or plateauing in the oxygen intake. The exercise test was stopped when the participants indicated that they were unable to continue with the exercise or once their systolic blood pressure reached 250 mmHg. The peak VO_2_ value (VO_2_peak, mL/min) was selected from the average oxygen intake values for every 30-s during the graded exercise. The VO_2_peak per FFM (mL/kg FFM/min) was used as the index of CRF.

### Statistical analysis

The participants were divided into two groups according to VF values by sex and age (low or high relative to the median VF value in 5-year increments). The participants were also divided into tertiles based on their CRF values by sex and age (in 5-year increments) and the lowest tertile was used as the reference. Continuous variables were reported as median values (interquartile range, IQR) and categorical variables were reported as numbers (percentages).

Logistic regression models were used to evaluate the relationships between VF or CRF and the prevalence of pre-DM/DM. After adjusting for confounding factors, the results were reported as the adjusted odds ratio (OR) and corresponding 95% confidence interval (95% CI). Model 1 was adjusted for various factors that are thought to be related to the onset of pre-DM/DM: age (continuous variable), sex (male/female), history of hypertension (yes/no), history of dyslipidemia (yes/no), smoking habit (current smoker, former smoker, or never smoker), energy intake (continuous variable), alcohol intake (continuous variable), and menstruation status (yes/no). Model 2 was adjusted for the Model 1 covariates plus VO_2_peak (continuous variable) to evaluate the relationship between VF and pre-DM/DM or VF (continuous variable) to evaluate the relationship between CRF and pre-DM/DM. Furthermore, we performed sub-group analyses according to VF grouping using pre-DM/DM (yes/no) as the dependent variable and CRF (tertile) as the independent variable in the logistic regression model. These statistical analyses were performed using SPSS Statistics software (version 25; IBM Corporation, Armonk, NY, USA).

A post-hoc power analysis was performed using the G*Power 3.1 [30] to evaluate whether our data had sufficient verification power.

## Results

The eligible patients included 643 men with a median age of 55 years (IQR: 48–64 years) and a median BMI of 23.3 kg/m^2^ (IQR: 21.6–25.1 kg/m^2^), as well as 327 women with a median age of 50 years (IQR: 45–57 years) and a median BMI of 21.0 kg/m^2^ (IQR: 19.4–22.8 kg/m^2^). Seventy-three participants had pre-DM and 48 participants had DM. None of the participants without pre-DM and DM were taking anti-diabetic drugs. Table 1 shows the physical characteristics of the groups with pre-DM/DM or no DM.

**Table 1:** Characteristics according to diabetes mellitus status.

	Total	No diabetes mellitus	Pre- and diabetes mellitus
Participants, n (male/female)	970 (643/327)	849 (541/308)	121 (102/19)
Age (years)	53 (47–62)	53 (46–60)	63 (55–68)
Height (cm)	166.8 (160.6–172.4)	166.5 (160.4–172.5)	169.0 (162.5–172.1)
Body weight (kg)	62.7 (55.4–71.0)	62.2 (54.8–70.2)	66.6 (59.6–75.0)
BMI (kg/m^2^)	22.5 (20.7–24.6)	22.4 (20.6–24.4)	23.6 (21.9–26.1)
% body fat (%)	22.5 (18.9–26.8)	22.7 (18.8–26.9)	21.8 (19.2–25.6)
FFM (kg)	50.0 (41.1–55.9)	49.5 (40.5–55.5)	52.2 (47.0–59.0)
Visceral fat area (cm^2^)	69.4 (43.6–101.0)	65.6 (41.8–94.8)	99.5 (69.6–133.2)
Subcutaneous fat area (cm^2^)	127.7 (91.4–170.4)	125.9 (90.7–170.2)	132.4 (95.1–171.4)
VO_2_peak/BW (mL/kg BW/min)	27.9 (24.4–32.2)	28.4 (24.6–32.5)	25.8 (22.2–29.2)
VO_2_peak/FFM (mL/kg FFM/min)	36.2 (32.3–41.0)	36.6 (32.7–41.4)	33.9 (29.3–37.2)
SBP (mmHg)	125.5 (113.0–138.0)	124.5 (112.5–136.0)	135.0 (123.3–148.3)
DBP (mmHg)	79.5 (72.0–88.0)	79.0 (72.0–87.5)	83.0 (74.5–92.5)
Hypertension, n (%)	352 (36.3)	277 (32.6)	75 (62.0)
Plasma glucose (mg/dL)	93 (88–100)	92 (87–97)	115 (110–125)
HbA_1c_ (%)	5.4 (5.2–5.6)	5.3 (5.1–5.5)	6.1 (5.8–6.5)
Insulin (μU/mL)	5.0 (3.6–7.6)	4.9 (3.5–7.3)	6.4 (4.1–9.1)
Diabetes mellitus, n (%)	48 (4.9)	0 (0.0)	48 (39.7)
Pre- diabetes mellitus, n (%)	73 (7.5)	0 (0.0)	73 (60.3)
Triglycerides (mg/dL)	79 (57–116)	76 (56–113)	97 (66–141)
Total cholesterol (mg/dL)	208 (187–234)	208 (186–233)	209 (191–238)
LDL-cholesterol (mg/dL)	121 (100–141)	121 (100–141)	121 (107–146)
HDL-cholesterol (mg/dL)	65 (54–76)	66 (55–76)	57 (47–70)
Dyslipidemia, n (%)	396 (40.8)	330 (38.9)	66 (54.5)
Energy intake (kcal/day)	1,907 (1,612–2,306)	1,899 (1,603–2,293)	2,027 (1,700–2,368)
Alcohol intake (g/day)	8.3 (0.6–25.2)	7.5 (0.5–24.1)	15.1 (1.4–31.1)
Current smoker, n (%)	63 (6.5)	49 (5.8)	14 (11.6)
Former smoker, n (%)	360 (37.1)	298 (35.1)	62 (51.2)
Never smoker, n (%)	547 (56.4)	502 (59.1)	45 (37.2)
Menstruating women, n (%)	171 (17.6)	166 (19.6)	5 (4.1)

BMI, body mass index; FFM, fat free mass; BW, body weight; SBP, systolic blood pressure; DBP, diastolic blood pressure; HbA1c, glycosylated hemoglobin; LDL, low-density lipoprotein; HDL, high-density lipoprotein.

Values are presented as median values (interquartile range) or numbers (%).

Compared to participants with no DM, participants with pre-DM/DM were older, had higher BMI and VF values, and had lower VO_2_peak values. Furthermore, participants with pre-DM/DM had higher blood pressure values, greater alcohol intake, and were more likely to be smokers. S1 Table shows the physical characteristics according to VF category and S2 Table shows the physical characteristics according to CRF tertile. Evaluations according to CRF tertile revealed no clear differences in age or BMI, although the highest CRF group had a lower percentage of body fat and VF than the other two groups. Table 2 shows the relationships between VF or CRF and the prevalence of pre- DM/DM.

**Table 2:** Odds ratios for the prevalence of pre-diabetes and diabetes mellitus according to visceral fat area or cardiorespiratory fitness categories.

		*n*	Number of cases	Model 1 ^a^	Model 2 ^b^
				OR (95% CI)	OR (95% CI)
**Visceral fat area**					
	Low VF	481	39	1.00 (reference)	1.00 (reference)
	High VF	489	82	2.04 (1.31–3.19)	1.87 (1.18–2.96)
**Cardiorespiratory fitness**					
	T1 (lowest)	319	52	1.00 (reference)	1.00 (reference)
	T2	327	38	0.68 (0.42–1.11)	0.74 (0.45–1.21)
	T3 (highest)	324	31	0.60 (0.36–1.00)	0.74 (0.44–1.26)
	*P* for trend			0.044	0.239

OR, odds ratio; CI, confidence interval; VF, visceral fat.

^a^ Model 1: adjusted for age, sex, hypertension, dyslipidemia, smoking habit, energy intake, alcohol intake, and menstruation status.

^b^ Model 2: adjusted for the Model 1 covariates plus VO_2_peak/FFM (for visceral fat area) or visceral fat area (for cardiorespiratory fitness).

After adjusting for potential risk factors (Model 1), and compared to the low VF group, the high VF group had a higher OR of pre-DM/DM (OR: 2.04, 95% CI: 1.31–3.19). After further adjustment for CRF (Model 2), the high VF group still had a higher OR of pre-DM/DM (OR: 1.87, 95% CI: 1.18–2.96). Although CRF was related to the prevalence of pre-DM/DM in Model 1 (Model 1, *P* for trend = 0.044), the relationship disappeared after additional adjusting for VF (Model 2, *P* for trend = 0.239). Compared to the lowest tertile for CRF, a slightly lower OR of pre-DM/DM prevalence was observed in the middle tertile for CRF (OR: 0.74, 95% CI: 0.45–1.21) and in the highest tertile for CRF (OR: 0.74, 95% CI: 0.44–1.26). Post-hoc power analyses were conducted which showed these sample sizes had more than 95% power to detect the association.

Sub-group analyses were also performed to evaluate whether the relationship between CRF and pre-DM/DM varied according to the VF classification (Table 3).

**Table 3:** Odds ratios for the prevalence of pre-diabetes and diabetes mellitus according to cardiorespiratory fitness categories in the visceral fat area sub-groups.

		Visceral fat area					
		Low			High		
		*n*	Number of cases	OR (95% CI)	*n*	Number of cases	OR (95% CI)
**Cardiorespiratory fitness**							
	T1 (lowest)	156	11	1.00 (reference)	160	36	1.00 (reference)
	T2	164	18	1.95 (0.83–4.59)	166	26	0.61 (0.33–1.12)
	T3 (highest)	161	10	1.01 (0.39–2.61)	163	20	0.48 (0.25–0.92)
	*P* for trend			0.979			0.024

OR, odds ratio; CI, confidence interval.

Adjusted for age, sex, hypertension, dyslipidemia, smoking habit, energy intake, alcohol intake, and menstruation status.

In the low VF group, CRF was not significantly related to the prevalence of pre-DM/DM (*P* for trend = 0.979). A post-hoc power analysis indicated a power of 5.9% in the low VF group. However, in the high VF group, CRF values were inversely associated with the prevalence of pre-DM/DM (*P* for trend = 0.024). Compared to the lowest CRF tertile, the lower OR of pre-DM/DM was observed in the middle CRF tertile (OR: 0.61, 95% CI: 0.33–1.12) and in the highest CRF tertile (OR: 0.48, 95% CI: 0.25–0.92). A post-hoc power analysis was conducted and found this sample size had a power of 95%.

## Discussion

This cross-sectional study evaluated the relationships of VF and CRF with pre-DM/DM among Japanese individuals. The results indicated no clear relationship between CRF and pre-DM/DM prevalence, although VF was associated with pre-DM/DM. However, sub-group analyses according to VF classification revealed that CRF values were inversely related to the prevalence of pre-DM/DM among individuals with high VF values.

Several cohort studies have evaluated the relationship between CRF per body weight (VO_2_max/BW) or BMI and the development of DM in Japanese individuals [13, 14, 31]. Kuwahara et al. [31] reported a higher risk of DM among men in the low CRF group, compared to the high CRF group, although this relationship disappeared after adjusting for BMI. Furthermore, a high BMI was associated with approximately double the risk of DM, compared to a low BMI, and this result was not substantially affected by adjusting for CRF. Their results suggested that the effect of CRF on DM was primarily mediated by body weight. In the present study, we used VO_2_peak/FFM as the index of CRF to reduce confounding by adiposity, and it was not significantly associated with pre-DM/DM, although the relationship with VF was significant; these results were similar to those of a previous study [31]. However, Kawakami et al. [14] performed a 23-year follow-up study of men and observed that higher baseline CRF values were associated with lower risks of DM development, and this relationship remained unchanged after adjustment for BMI. The discrepancy between their findings and our findings may be related to the timing of CRF assessment (e.g., baseline CRF values vs CRF values at follow up). A recent meta-analysis has also evaluated the relationship between CRF and the risk of type 2 DM [16], which revealed that high CRF values and low BMI values were associated with a low risk of type 2 DM, although low BMI was the more influential factor. Several previous cohort studies have also revealed similar results regarding the effects of CRF adjusted for body weight and BMI on the risk of developing DM, which suggests that BMI plays a greater role than CRF, even when differences in the BMI cut-off point, method of fitness assessment, and follow-up duration were considered. Thus, controlling body weight may be more important for preventing type 2 DM than improving CRF.

Many researchers have reported that DM onset occurs among Asians at lower BMI levels than among Western populations [2, 6–8]. Furthermore, when comparing individuals with the same BMI, Asians have more VF than populations of European descent [7, 9, 10]. Thus, relative to Western populations, abdominal adiposity in Asians may play a bigger role in DM development than BMI, which is related to physical stature. Previous studies of Asians have also indicated that abdominal adiposity plays a more significant role in DM and insulin resistance [10, 18, 32, 33]. The present study aimed to address this issue by using VO_2_peak/FFM as the CRF index, rather than VO_2_peak/BW to avoid confounding from obesity. Our results were consistent with the results from our previous study, which indicated that VF, but not CRF (VO_2_max/BW), was involved in insulin resistance [12].

Our sub-group analyses according to VF classification revealed that higher CRF values were inversely associated with DM prevalence among individuals in the high VF group. It is unclear how differences in VF classification might influence the relationship between CRF and pre-DM/DM prevalence, although we suspect that differences in CRF and accumulated ectopic fat might explain the results in the high VF group. Granados et al. [34] have reported that higher abdominal intermuscular adipose tissue (IMAT) volume, based on computed tomography, was associated with a higher prevalence of pre-DM/DM, and that this association was closely related to the VF value. Furthermore, the IMAT volume was inversely proportional to aerobic physical fitness quantified based on total treadmill duration [34]. A previous study of non-obese Asians revealed that VF and intrahepatic triglycerides (IHTGs), which were measured using magnetic resonance spectroscopy, were inversely correlated to insulin sensitivity [35] and that VF and IHTGs were inversely proportional to CRF (VO_2_max/BW). These results suggest that the deposition of ectopic fat might be suppressed in individuals with high CRF and high VF levels. Thus, even in individuals with high VF values, improving CRF and metabolizing ectopic fat via high-intensity aerobic physical activity may help control the onset of DM.

This study has several limitations. The first limitation is the cross-sectional design, which precludes a conclusion regarding the causality of the relationship between VF/CRF and pre-DM/DM. Nevertheless, the results may help guide interventions that aim to limit the risk of developing pre-DM/DM according to the participant’s physical condition and VF level. The second limitation is the relatively small sample size of the low VF group for sub-group analyses. It is important to consider a power of analysis, especially when dealing with a small number of subjects indicating low prevalence. We need to increase the sample size and to reanalyze the association between prevalence of pre-DM/DM and CRF (VO2max/FFM) in participants with low VF. The third limitation is the inclusion of men and women, although we adjusted the analyses for sex and menstruation status. In this context, men and women have different fat distributions [36] and women experience variations in their lipid metabolism according to menstruation status [37]. Nevertheless, we did not detect significant interactions between sex and CRF (*P* for interaction = 0.597) or VF (*P* for interaction = 0.418) on pre-DM/DM prevalence.

## Conclusions

The present study failed to detect an independent relationship between CRF and pre-DM/DM after adjusting for adiposity. However, higher VF values were related to a higher prevalence of pre-DM/DM. Furthermore, especially among individuals with high VF values, higher CRF values were associated with a lower prevalence of pre-DM/DM.

## Supporting information

S1 Table Characteristics according to visceral fat area category. VF, visceral fat; BMI, body mass index; FFM, fat free mass; BW, body weight; SBP, systolic blood pressure; DBP, diastolic blood pressure; HbA1c, glycosylated hemoglobin; LDL, low-density lipoprotein; HDL, high-density lipoprotein. Values are presented as medians (interquartile range) or numbers (%).
(DOCX)

S2 Table Characteristics according to cardiorespiratory fitness category. BMI, body mass index; FFM, fat free mass; BW, body weight; SBP, systolic blood pressure; DBP, diastolic blood pressure; HbA1c, glycosylated hemoglobin; LDL, low-density lipoprotein; HDL, high-density lipoprotein. Values are presented as medians (interquartile range) or numbers (%).
(DOCX)
