# Supplementary material for: Visceral fat and cardiorespiratory fitness with prevalence of pre-diabetes/diabetes mellitus among middle-aged and elderly Japanese people: WASEDA’S Health Study
Source: PLoS One. 2020 Oct 20;15(10):e0241018. doi: 10.1371/journal.pone.0241018 (PMC7575118; doi:10.1371/journal.pone.0241018)
Supplement: S1 Table — VF, visceral fat; BMI, body mass index; FFM, fat free mass; BW, body weight; SBP, systolic blood pressure; DBP, diastolic blood pressure; HbA1c, glycosylated hemoglobin; LDL, low-density lipoprotein; HDL, high-density lipoprotein. Values are presented as medians (interquartile range) or numbers (%). (DOCX) [file pone.0241018.s001.docx]

**S1 Table.** **Characteristics according to visceral fat area category**

|  | Low VF | | High VF | |
| --- | --- | --- | --- | --- |
| Participants, n (male/female) | 481 (319/162) | | 489 (324/165) | |
| Age (years) | 53 | (47–62) | 54 | (47–62) |
| Height (cm) | 166.8 | (160.2–172.2) | 166.7 | (161.0–172.6) |
| Body weight (kg) | 59.6 | (52.5–65.9) | 66.9 | (58.8–75.2) |
| BMI (kg/m^2^) | 21.4 | (19.9–22.9) | 23.9 | (22.1–25.9) |
| % body fat (%) | 19.6 | (16.4–23.4) | 24.9 | (21.8–29.1) |
| FFM (kg) | 48.6 | (40.5–54.0) | 51.4 | (41.4–57.8) |
| Visceral fat area (cm^2^) | 46.4 | (30.5–65.7) | 100.9 | (76.6–125.8) |
| Subcutaneous fat area (cm^2^) | 97.0 | (72.4–131.2) | 155.6 | (123.7–194.9) |
| VO_2_peak/BW (mL/kg BW/min) | 30.1 | (26.1–35.0) | 26.2 | (22.6–30.0) |
| VO_2_peak/FFM (mL/kg FFM/min) | 37.4 | (33.5–43.1) | 35.3 | (31.1–39.3) |
|  |  |  |  |  |
| SBP (mmHg) | 122.0 | (110.5–135.3) | 128.5 | (116.5–141.0) |
| DBP (mmHg) | 77.5 | (70.0–85.3) | 82.0 | (74.8–90.8) |
| Hypertension, n (%) | 136 | (28.3) | 216 | (44.2) |
| Plasma glucose (mg/dL) | 92 | (87–98) | 95 | (89–103) |
| HbA_1c_ (%) | 5.3 | (5.1–5.6) | 5.4 | (5.2–5.7) |
| Insulin (μU/mL) | 4.1 | (3.1–5.6) | 6.5 | (4.6–9.0) |
| Diabetes mellitus, n (%) | 15 | (3.1) | 33 | (6.7) |
| Pre-diabetes mellitus, n (%) | 24 | (5.0) | 49 | (10.0) |
| Triglycerides (mg/dL) | 65 | (51–88) | 96 | (71–142) |
| Total cholesterol (mg/dL) | 207 | (188–234) | 210 | (187–234) |
| LDL-cholesterol (mg/dL) | 118 | (98–138) | 123 | (104–146) |
| HDL-cholesterol (mg/dL) | 69 | (59–80) | 59 | (50–70) |
| Dyslipidemia, n (%) | 144 | (36.4) | 252 | (51.5) |
|  |  |  |  |  |
| Energy intake (kcal/day) | 1,902 | (1,619–2,266) | 1,915 | (1,607–2,355) |
| Alcohol intake (g/day) | 5.3 | (0.2–20.5) | 10.3 | (1.1–31.0) |
| Current smoker, n (%) | 17 | (3.5) | 46 | (9.4) |
| Former smoker, n (%) | 166 | (34.5) | 194 | (39.7) |
| Never smoker, n (%) | 298 | (62.0) | 249 | (50.9) |
| Menstruating women, n (%) | 89 | (18.5) | 82 | (16.8) |

VF, visceral fat; BMI, body mass index; FFM, fat free mass; BW, body weight; SBP, systolic blood pressure; DBP, diastolic blood pressure; HbA1c, glycosylated hemoglobin; LDL, low-density lipoprotein; HDL, high-density lipoprotein.

Values are presented as medians (interquartile range) or numbers (%).
