# Supplementary material for: Visceral fat and cardiorespiratory fitness with prevalence of pre-diabetes/diabetes mellitus among middle-aged and elderly Japanese people: WASEDA’S Health Study
Source: PLoS One. 2020 Oct 20;15(10):e0241018. doi: 10.1371/journal.pone.0241018 (PMC7575118; doi:10.1371/journal.pone.0241018)
Supplement: S2 Table — BMI, body mass index; FFM, fat free mass; BW, body weight; SBP, systolic blood pressure; DBP, diastolic blood pressure; HbA1c, glycosylated hemoglobin; LDL, low-density lipoprotein; HDL, high-density lipoprotein. Values are presented as medians (interquartile range) or numbers (%). (DOCX) [file pone.0241018.s002.docx]

**S2 Table.** **Characteristics according to cardiorespiratory fitness category**

|  | T1  (lowest) | T2 | T3  (highest) |
| --- | --- | --- | --- |
| Participants, n (male/female) | 319 (213/106) | 327 (216/111) | 324 (214/110) |
| Age (years) | 54.0 (47.0–62.0) | 53.0 (47.0–62.0) | 53.0 (47.0–60.8) |
| Height (cm) | 167.8 (161.1–172.9) | 166.6 (160.6–172.0) | 166.3 (160.2–172.5) |
| Body weight (kg) | 63.6 (56.1–74.5) | 63.7 (55.3–70.4) | 61.2 (54.6–69.2) |
| BMI (kg/m^2^) | 22.8 (20.7–25.3) | 22.6 (20.9–24.8) | 22.2 (20.6–23.9) |
| % body fat (%) | 23.7 (20.1–27.9) | 22.8 (19.6–26.6) | 20.7 (17.0–25.5) |
| FFM (kg) | 49.8 (40.4–57.0) | 50.4 (41.4–55.0) | 49.8 (41.4–55.6) |
| Visceral fat area (cm^2^) | 81.1 (52.5–114.7) | 75.5 (46.5–103.1) | 57.8 (36.8–85.1) |
| Subcutaneous fat area (cm^2^) | 134.1 (98.9–184.2) | 132.9 (98.5–173.9) | 111.3 (82.6–152.5) |
| VO_2_peak/BW (mL/kg BW/min) | 23.3 (20.7–26.2) | 28.0 (25.5–30.6) | 33.6 (30.1–37.5) |
| VO_2_peak/FFM (mL/kg FFM/min) | 30.9 (27.8–33.8) | 36.7 (34.0–38.9) | 43.0 (39.7–46.8) |
|  |  |  |  |
| SBP (mmHg) | 126.0 (116.0–139.5) | 124.5 (112.5–139.0) | 125.5 (112.5–136.0) |
| DBP (mmHg) | 80.5 (72.5–89.5) | 79.5 (72.5–88.5) | 78.5 (71.0–87.0) |
| Hypertension, n (%) | 130 (40.8) | 122 (37.3) | 100 (30.9) |
| Plasma glucose (mg/dL) | 94 (89–101) | 93 (88–100) | 93 (88–99) |
| HbA_1c_ (%) | 5.4 (5.2–5.7) | 5.4 (5.2–5.6) | 5.4 (5.2–5.6) |
| Insulin (μU/mL) | 5.5 (3.6–8.1) | 5.1 (3.6–7.5) | 4.6 (3.5–6.6) |
| Diabetes mellitus, n (%) | 25 (7.8) | 12 (3.7) | 11 (3.4) |
| Pre-diabetes mellitus, n (%) | 27 (8.5) | 26 (8.0) | 20 (6.2) |
| Triglycerides (mg/dL) | 84 (58–125) | 84 (62–115) | 71 (54–105) |
| Total cholesterol (mg/dL) | 204 (184–231) | 209 (186–231) | 211 (191–239) |
| LDL-cholesterol (mg/dL) | 116 (96–141) | 122 (102–141) | 122 (102–143) |
| HDL-cholesterol (mg/dL) | 63 (51–73) | 63 (54–75) | 67 (56 – 80) |
| Dyslipidemia, n (%) | 138 (43.3) | 134 (41.0) | 124 (38.3) |
|  |  |  |  |
| Energy intake (kcal/day) | 1,847 (1,562–2,270) | 1,940 (1,665–2,326) | 1,927 (1,637–2,324) |
| Alcohol intake (g/day) | 7.5 (0.2–27.4) | 10.8 (0.7–27.7) | 6.5 (0.6–21.9) |
| Current smoker, n (%) | 23 (7.2) | 21 (6.4) | 19 (5.9) |
| Former smoker, n (%) | 108 (33.9) | 142 (43.4) | 110 (34.0) |
| Never smoker, n (%) | 188 (58.9) | 164 (50.2) | 195 (60.2) |
| Menstruating women, n (%) | 55 (17.2) | 56 (17.1) | 60 (18.5) |

BMI, body mass index; FFM, fat free mass; BW, body weight; SBP, systolic blood pressure; DBP, diastolic blood pressure; HbA1c, glycosylated hemoglobin; LDL, low-density lipoprotein; HDL, high-density lipoprotein.

Values are presented as medians (interquartile range) or numbers (%).
